# Interfacial Segregation of Sn during the Continuous Annealing and Selective Oxidation of Fe-Mn-Sn Alloys

**DOI:** 10.3390/ma17061257

**Published:** 2024-03-08

**Authors:** Jonas Wagner, Joseph R. McDermid

**Affiliations:** McMaster Steel Research Centre, Department of Materials Science and Engineering, McMaster University, 1280 Main Street West, Hamilton, ON L8S 4L8, Canada; wagnej5@mcmaster.ca

**Keywords:** Sn microalloying, interfacial segregation, selective oxidation, continuous annealing, atom probe tomography, auger electron spectroscopy

## Abstract

The effect of Mn on interfacial Sn segregation during the selective oxidation of Fe-(0–10)Mn-0.03Sn (at.%) alloys was determined for annealing conditions compatible with continuous galvanizing. Significant Sn enrichment was observed at the substrate free surface and metal/oxide interface for all annealing conditions and Mn levels. Sn enrichment at the free surface was insensitive to the Mn alloy concentration, which was partially attributed to the opposing effects of Mn on segregation thermodynamics and kinetics: Mn increases the driving force for Sn segregation via reducing Sn solubility in Fe but also reduces the effective Sn diffusivity by increasing the austenite volume fraction. This insensitivity was exacerbated by the depletion of solute Mn near the surface due to the selective oxidation of Mn. Thus, Sn segregation occurred in regions with a local Mn concentration lower than the nominal bulk composition of the alloys suggested. Sn enrichment at the metal/external oxide interface was reduced compared to the free surface and decreased with increasing bulk Mn content, which was attributed to changes in the external oxide morphology and metal/internal oxide interfaces acting as Sn sinks.

## 1. Introduction

Sn is a common residual element in steel [1,2] which is well known to segregate to external surfaces and internal interfaces such as grain boundaries (GBs) at high temperatures. Models for equilibrium segregation have been developed within the context of temper embrittlement [3,4,5], where the enrichment of impurities at GBs is generally seen as undesirable. More recently, “segregation engineering” has become a tool for tailoring the design of interfacial properties [6,7], and deliberate Sn micro-additions to advanced high strength steels (AHSSs) have been suggested to improve the quality of galvanized coatings [8,9,10,11]. In continuous galvanizing lines (CGLs), commonly employed annealing atmospheres suppress Fe oxidation, while less noble alloying additions—such as Al, Cr, Mn, and Si—undergo selective oxidation. A thick, continuous layer of external oxides is highly detrimental to reactive wetting of the strip in the molten Zn(Al) bath and will often result in unacceptable coating quality and poor adhesion of the Zn overlay [12].

When surface active elements such as Sn, Sb, and Bi are added to the steel, they are known to segregate to the surface during annealing and decrease the kinetics of external and internal oxidation [8,9,10,11,13,14]. This has been attributed to segregant atoms reducing the oxygen supply by occupying oxygen adsorption sites, as observed for gas–metal reactions such as carburizing [15]. The segregation of surface active elements can also cause morphological changes from film-type to island-type external oxides, arising from the modification of the surface energy balance [9].

One of the principal alloying elements in Third Generation AHSS (3G AHSS) is Mn, with concentrations up to 10 at.% [16,17]. Besides increasing solid solution strengthening, Mn stabilizes austenite and lowers the martensite start and finish temperatures, thereby promoting the retention of austenite in these medium Mn (med-Mn) transformation induced plasticity (TRIP)-assisted 3G AHSSs. The subsequent deformation-induced transformation of the metastable retained austenite to martensite maintains high work hardening rates, thereby enhancing the alloy plasticity whilst increasing the mechanical properties [16,18]. In order for Sn microalloying to be effective, significant Sn surface coverage must be established within the short isothermal annealing times characteristic of the CGL, commonly between 60 and 120 s [18]. Thus, it is important to understand how the Mn level of the steel affects the interfacial segregation of Sn and how this interacts with the Zn-based liquid alloy in the CGL bath.

The effect of a 0.02 at.% Sn addition has been explored in recent studies. For example, the observed Sn enrichment ratio was 130 in a dual-phase (DP) steel with 2 at.% Mn [11], but it was only 10 in a med-Mn steel with 6 at.% Mn [10]. However, a direct comparison of these results is difficult, as different annealing temperatures and processing atmosphere oxygen partial pressures (i.e., pO_2_) were employed. Currently, no systematic investigation of Sn enrichment in substrates representative of the Mn contents typical of 3G AHSSs is available. Thus, the objective of this study is to determine the interaction between Mn and Sn and its effects on the interfacial segregation of Sn during CGL-compatible annealing, with the ultimate objective of evaluating the applicability of Sn microalloying to improve the galvanizing performance of Mn-containing AHSSs.

## 2. Materials and Methods

Four sets of Fe-Mn-Sn model alloys (0.08 at.% C, 0.008 at.% Ni, 0.003 at.% P, 0.016 at.% Si, 0.018 at.% Cu) were fabricated by induction melting under a protective Ar atmosphere. Raw materials for alloy fabrication comprised ARMCO Grade 4 Fe (99.9% Fe), electrolytic Fe (North American Höganäs High Alloys LLC, Johnstown, PA, USA)–Grade A-276 (99.97% purity), electrolytic Mn (Belmont Metals Inc., Brooklyn, NY, USA, 99.9% Mn), and Sn shot (Alpha Aesar, Ward Hill, MA, USA, 99.999% Sn). Sn was added to the alloys via a Fe-10 wt.% Sn master alloy fabricated from the electrolytic Fe and Sn shot. The bulk alloys were fabricated using the Armco Fe, electrolytic Mn, and the Fe-10Sn (wt.%) master alloy. The compositions of the experimental alloys, as determined using inductively coupled plasma optical emission spectroscopy (ICP-OES), are shown in Table 1. The Sn bulk concentration of 0.03 at.% was selected for the model Fe-Mn-Sn alloys used in this study to ensure significant interfacial segregation during the relatively short isothermal holding times characteristic of continuous galvanizing, as discussed above, while remaining below Sn concentrations of 0.1 wt.% (~0.05 at.%), known to be detrimental to alloy castability and hot forming [1].

**Figure 1:**
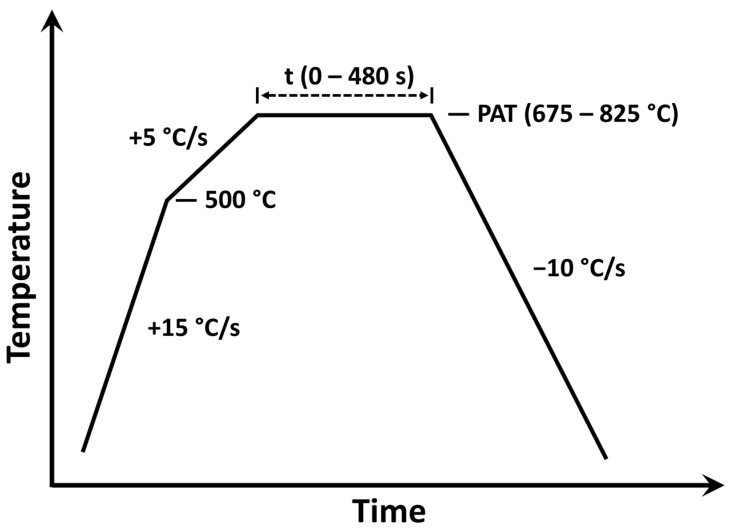
Schematic of experimental annealing heat treatment.

The as-cast ingots were homogenized for 24 h at 1000 °C and hot rolled to a thickness of 4 mm. After descaling via a 16 vol. % hydrochloric acid solution inhibited by hexamethylenetetramine, the hot rolled ingots were cold rolled to 1.2 mm thickness and cut into 20 × 50 mm coupons. To reduce the effect of rolling-induced roughness on surface analysis techniques, samples were ground to a 1200 grit finish with SiC paper. Heat treatments were conducted in the McMaster Galvanizing Simulator (Iwani-Surtec, Düsseldorf, Germany) [19] under a flowing N_2_-5vol%H_2_ process atmosphere with a dew point of −10 °C. A schematic of the heat treatment cycle is shown in Figure 1 and the corresponding annealing conditions are stated in Table 2. The annealing parameters were chosen to include a typical 3G AHSS CGL-compatible processing window [18] in order to determine the amount of interfacial Sn segregation observed during continuous annealing. The pO_2_ of the annealing atmosphere ranged between 9.2 × 10^−25^ atm at 675 °C and 5.0 × 10^−21^ atm at 825 °C (Table 2). This is below the equilibrium pO_2_ for forming Fe or Sn oxides but above that for forming MnO [20], suggesting that there was a significant driving force for the selective oxidation of Mn during annealing.

The evolution of Sn segregation at external and internal interfaces as a function of annealing time, temperature, and Mn content were investigated by several complementary techniques. Sn segregation to the external surface was measured by Auger Electron Spectroscopy (AES) in a JAMP-9500F field-emission scanning Auger microscope (i.e., FEG-SAM, JEOL, Tokyo, Japan). An acceleration voltage of 10 kV and a primary beam current of 80 nA was used for all analyses in order to enable the ca. 60 nm lateral resolution required to analyze the surface between the Mn-oxide nodules that formed during annealing. Gentle sputtering with Ar^+^ ions between spectral acquisitions was performed to reduce the effect of adsorbed hydrocarbons on the intensity of the Sn signal. For each sample, the concentration of segregated Sn was quantified from the spectrum with maximum relative Sn intensity using Spectra Investigator v1.09 (JEOL Ltd.) software.

To qualitatively screen metal/oxide interfaces of external and internal oxides for Sn enrichment, lift outs for transmission electron microscopy (TEM) were fabricated using focused ion beam (FIB) milling (ZEISS NVision 40, Oberkochen, Germany). Elemental maps were acquired by energy dispersive X-ray spectroscopy (EDS) using a Talos 200X TEM (ThermoScientific, Waltham, MA, USA) operated using a 200 kV acceleration voltage.

To quantify the interfacial Sn enrichment, 3D atom probe tomography (APT) was performed. FIB milling (Zeiss NVision 40, Thermo Fisher Helios 5 UC) was used to extract the specimens from the as-annealed sample surface, sharpen them into needles, and remove beam damage through low voltage cleaning [21]. Before lift out, a ~200 nm thick protective Cr or Cu coating was applied to preserve the external surface of the specimen. Data were acquired in laser pulsing mode (λ = 355 nm, laser pulse energy 60 pJ) at a base temperature of 60 K using a LEAP 4000X HR or LEAP 5000 XS instrument (Cameca Instruments, Fitchburg, WI, USA) with detection efficiencies of 0.36 and 0.8, respectively. The laser pulse rate for each run was initially set to 125 kHz and was increased throughout the experiment to optimize acquisition speed. The reconstructed data were analyzed using IVAS v6.1.3 software.

Two complementary metrics derived from the APT data were used to quantify interfacial segregation and compare Sn enrichment across different interface types and annealing conditions. The first metric was the maximum Sn concentration determined by a proximity histogram across the interface, based on the 50 at.% Fe iso-concentration surface. This methodology has the advantage of reducing the effect of interface topography and enables the calculation of a single average value across a curved metal/oxide interface. It also allows for direct comparison to the maximum concentration determined by SAM+AES.

The second metric was the interfacial excess (Γ) in atoms/nm^2^. Γ is a rigidly defined thermodynamic variable [22] which is less susceptible to the effects of the spatial resolution of the interface [23] in which the segregant concentration is normalized with respect to surface area. The degradation in spatial resolution can be quantified via a local decrease in atomic density within the interfacial region and as a broadening of the interface in the direction the sample was evaporated [24]. This degradation is caused by trajectory aberrations due to the materials adjacent to the heterointerface evaporating at a different field [24] and can be exacerbated by geometric factors such as distance from a crystallographic pole [25] or orientation of the interface with respect to acquisition direction [26]. To improve the accuracy of the determined Γ, the analyzed volume was extracted perpendicular to a planar subregion of the interface [27]. Thus, Γ, in the present case, is a more localized metric of segregation as compared to the maximum Sn concentration. Furthermore, by using the integral method described by Maugis and Hoummada [23], the measurement of Γ is independent of the positioning of a Gibbs dividing surface at the hard-to-define location of the interface. In this case, a 1D Sn concentration profile was measured perpendicular to the interface and was integrated over the width of the interfacial region according to the following:

(1) $$\Gamma =\int_{x_{1}}^{x_{2}}\left(C_{Sn}(x)-C_{Sn}^{ref}(x)\right)dx$$

where *C_Sn_* is the locally measured concentration of Sn in atoms/nm^3^, adjusted for the detection efficiency of the instrument [28], and *x*_1_ and *x*_2_ are the coordinates defined by the length of the interfacial region in the concentration profile (as indicated by the arrows in the APT elemental maps, an example of which is provided in Figure 6). A Boltzman sigmoid function fitted to Sn concentrations outside of the interfacial region [29] was used to determine the local reference concentration, $C_{Sn}^{ref}$. The Sn signal used to calculate these two metrics was based on the peaks of ^116^Sn^2+^, ^117^Sn^2+^, and ^119^Sn^2+^, where no peak overlap was observed for any sample and the signal was corrected for natural isotopic abundance [30].

To relate the amount of segregated Sn to the subsurface morphology of the Mn oxides, cross-sectional micrographs of the samples analyzed by APT were acquired on a Zeiss Crossbeam 350 FIB-SEM. A protective W coating was deposited prior to milling with Ga ions to protect the external oxides from beam damage. The length of the internal metal/oxide interfaces was subsequently measured in ImageJ v1.53g.

## 3. Results

### 3.1. Sn Segregation to the Free (External) Surface

**Figure 2:**
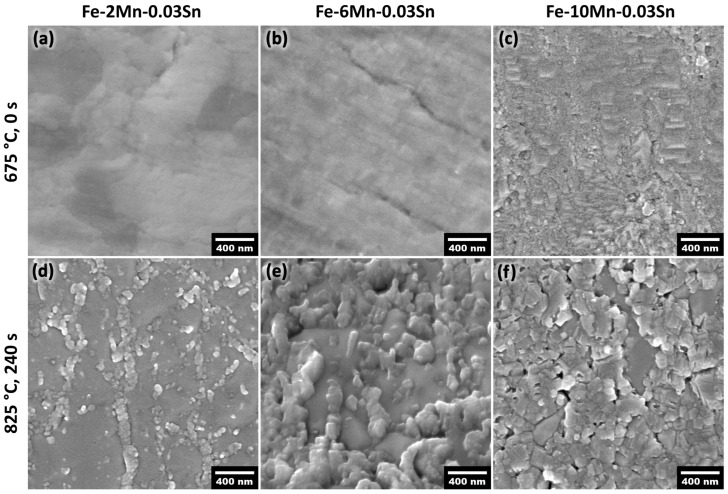
SEM micrographs of the surface of the Fe-xMn-0.03Sn alloys with Mn contents of (**a**,**d**) 2 at.%, (**b**,**e**) 6 at.%, and (**c**,**f**) 10 at.% after annealing for (**a**–**c**) 0 s at 675 °C (i.e., after linear heating) and (**d**–**f**) 240 s at 825 °C.

Mn was selectively oxidized during isothermal annealing while only a few small oxide nodules were formed on the surface during linear heating (Figure 2a–c). The external oxides coalesced into patches covering significant area fractions of the surface with increasing isothermal holding time and temperature (Figure 2d–f). The oxide coverage increased with increasing Mn alloy concentration for a given annealing time and temperature (Figure 2).

**Figure 3:**
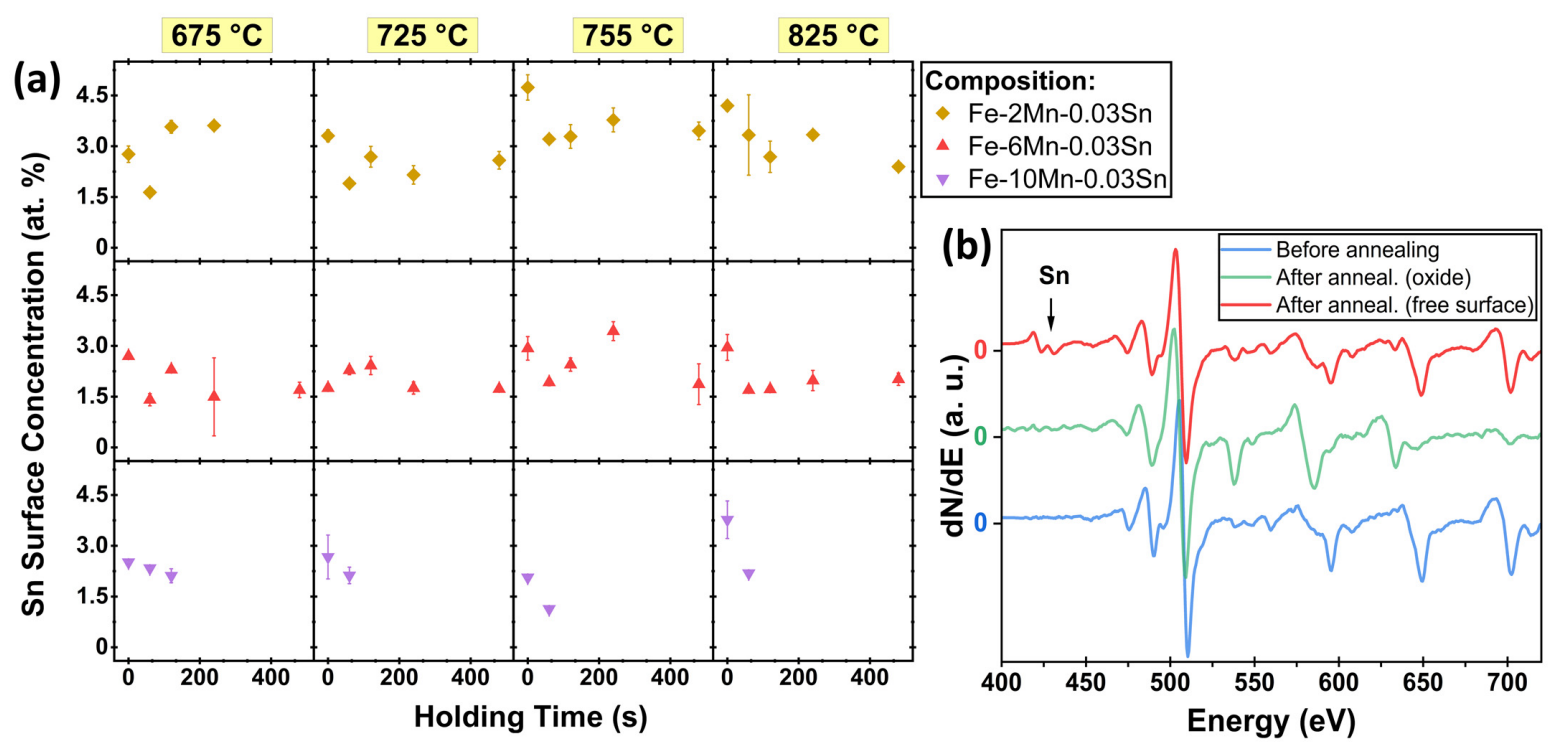
Sn segregation to the free surface during annealing. (**a**) Sn concentration on the free surface between oxide nodules of Fe-(2–10)Mn-0.03Sn (at.%) alloys after annealing for 0–480 s at 675–825 °C, as determined by AES. (**b**) Representative Auger spectra before and after annealing (measured on Mn oxide and on free surface between oxide nodules), in this case, for Fe-6Mn-0.03Sn after 240 s at 825 °C.

Segregation of Sn to the external surface was characterized by AES. Typical Auger spectra are shown in Figure 3b. After annealing, Sn MNN peaks (415–437 eV), O KLL peaks (475–510 eV), and Fe LMM peaks (554–730 eV) were clearly observed in the AES spectra acquired from the free surface between oxide nodules. When acquired from surface nodules, the O KLL and Mn LMM (542–636 eV) peaks dominated the spectrum, confirming that the nodules observed in Figure 2 were Mn oxides, as expected [10,14,19]. No Sn signal was detected on the sample surface before annealing or the external surface of the oxide nodules. Thus, the observed Sn enrichment was established exclusively during annealing; any Sn segregated during sample fabrication was removed in the sample preparation process. Furthermore, Sn was enriched on the surfaces between external oxides, with no indication of Sn being present on top of the oxide.

Figure 3a shows the surface concentration of Sn for the Fe-(2–10)Mn-0.03Sn (at.%) systems after annealing at 675–825 °C for 0–480 s, as determined by AES. The concentration was measured on the free surface between oxide nodules. No data are shown for annealing conditions for which the oxide coverage was too high to target the internodular space, in particular the Fe-10Mn-0.03Sn alloy after annealing for longer than 120 s. Where accessible, significant Sn segregation was observed for all annealing conditions and compositions investigated (Figure 3a). Sn enrichment ratios between the surface and bulk concentration ranged between 37 and 157. No trends with respect to isothermal holding times between 0 and 480 s could be observed, where significant Sn surface enrichment was observed at the onset of isothermal holding for all annealing temperatures (Figure 3a). This is consistent with earlier work on binary Fe-0.03Sn and Fe-0.01Sn alloys in which Sn enrichment for CGL-compatible annealing cycles was primarily established during the linear heating segment between 500 °C and 675 °C [31]. Furthermore, no significant differences in Sn surface concentration as a function of Mn content were observed—i.e., Sn surface segregation was insensitive to the Mn level for the annealing conditions investigated (Figure 3a).

### 3.2. Sn Segregation to the Metal/Oxide Interfaces

**Figure 4:**
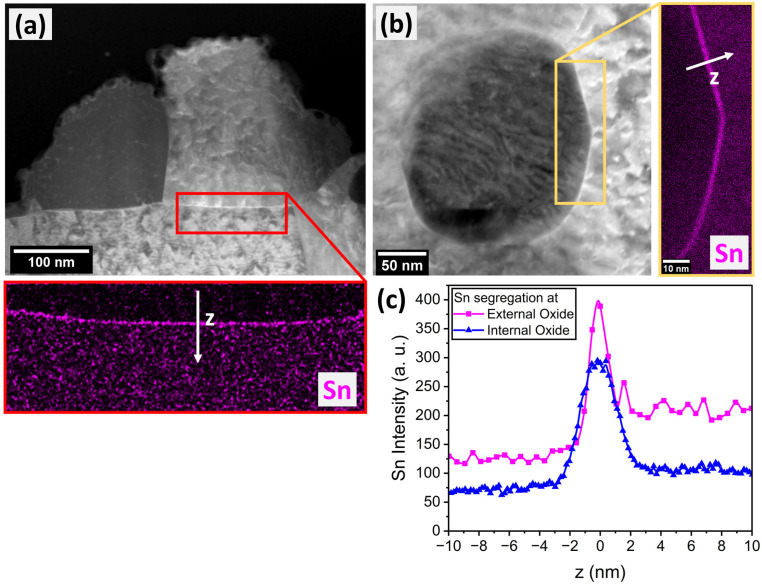
Sn segregation to metal/oxide interfaces. Cross-sectional STEM micrographs and EDS elemental Sn maps at the metal/oxide interface of (**a**) external and (**b**) internal oxide nodule. (**c**) Sn intensity profile across interfaces along white arrows shown in (**a**,**b**).

With increasing annealing temperature, holding time, and substrate Mn content, more of the sample surface was covered by oxide nodules, thereby converting an increasing fraction of the free substrate surface into metal/oxide interfaces (Figure 2). Such metal/oxide interfaces were characterized by TEM. Figure 4 shows the cross section of an external oxide nodule and a larger spherical internal oxide particle embedded in the metal matrix. Elemental mapping by EDS revealed the presence of a thin layer of Sn at the metal/oxide interface (Figure 4a,b). The apparent width of the Sn enrichment zone is approximately 2–3 nm, as approximated by the FWHM of the Sn intensity peak at the interface (Figure 4c).

**Figure 5:**
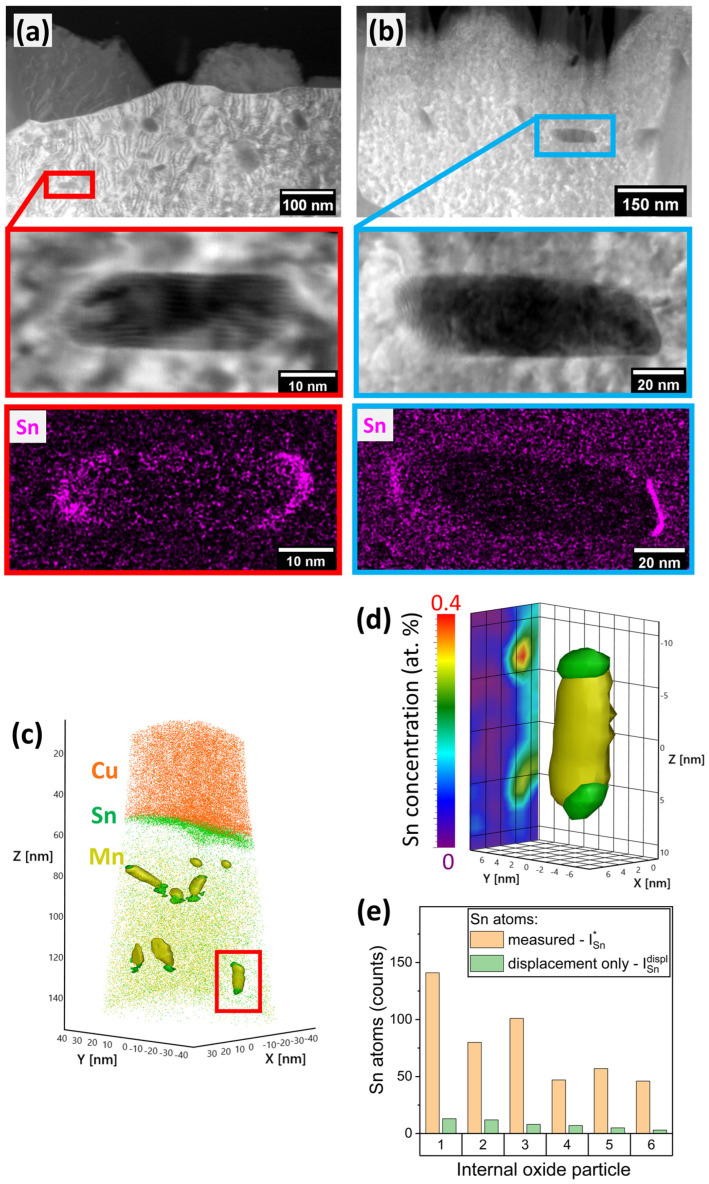
Sn segregation to small internal oxide particles. (**a**,**b**) STEM micrographs and EDS elemental Sn maps of small internal oxide particles found in the Fe-6Mn-0.03Sn alloy after 480 s at 825 °C. (**c**) APT reconstruction of a Fe-6Mn-0.03Sn specimen after 120 s at 675 °C. Internal oxide particles and Sn enrichment around the oxide particle vertices are shown by 10 at.% Mn and 0.4 at.% Sn iso-concentration surfaces. (**d**) Enlarged detail of internal oxide particle shown in the red box in (**a**), with Sn concentration projected on the YZ plane, (**e**) measured Sn atom enrichment around oxide vertices compared to the expected number of Sn atoms when only displacement of Sn by oxide particles is considered. All internal oxide particles from (**a**) with a volume of at least 100 nm^3^ were included.

STEM micrographs and EDS elemental Sn maps of two smaller, elongated internal oxide particles are shown in Figure 5a,b. Preferential interfacial segregation of Sn to the vertex regions of the particles was observed—i.e., higher Sn concentrations were detected at the short interfaces of the particle than on the elongated sides. Due to the finite thickness of the TEM sample, the apparent sharpness of the interface is affected by the curvature of the interface throughout the sample and the crystallographic orientation of the particle with respect to the matrix and the incident electron beam [32,33]. APT, which is based on sequential field evaporation of the specimen rather than electron–matter interactions—as is the case for the SEM and TEM—produces a 3D atom-by-atom model of the sample and enables a more detailed characterization of any curved internal interfaces. Figure 5c shows a reconstructed APT dataset of a specimen in which comparable small elongated internal oxide particles were found. For better visibility, the Fe matrix atoms are not shown. Segregation of Sn to the external surface (here observed as the interface with Cu, which was used as a protective coating) is apparent (Figure 5c). Internal oxides and adjacent Sn enrichment zones are represented by 10 at.% Mn and 0.4 at.% Sn iso-concentration surfaces, respectively.

Eight internal oxide particles were found, six of which had a volume of at least 100 nm^3^. For all elongated particles, Sn enrichment was observed in one or both of the vertex regions of each particle (Figure 5c), a magnified example of which is shown in Figure 5d. The presence of Sn in both vertex regions for some particles and in vertex regions of differently oriented particles with respect to the direction of evaporation (*z*-axis in Figure 5c) suggests that the observed spatial relation between the oxide particle and Sn enrichment zone is not an artefact of preferential retention [34] due to the different elemental evaporation fields in the APT specimen.

The preferential Sn segregation to the vertex region can be seen as evidence for equilibrium segregation being the governing mechanism for Sn enrichment at metal/internal oxide interfaces. Within this framework, an important contribution to the driving force for segregation is the reduction in the overall interfacial energy [35,36]. Here, the morphology of the internal oxide particles hints at the presence of higher-energy interfaces: based on classic nucleation theory, nucleation and growth of small particles are governed by the balance of free energy reduction by the completion of a thermodynamically favorable chemical reaction (in this case the oxidation of Mn) and the increase in free energy by the creation of interfaces [37,38]. The smaller the particle, the higher its surface-to-volume ratio and the stronger the contribution of the surface energy term to the selection of the particle’s growth direction. From the morphology of the oxides, it can be deduced that the interfaces parallel to the elongated direction of the particles are associated with lower interfacial energy. By way of contrast, the interfaces at the vertices, where preferential segregation is observed, possess higher interfacial energy. Thus, the increased interfacial segregation associated with higher interfacial energy supports the suggested equilibrium segregation mechanism. In addition to equilibrium segregation, the displacement of dissolved Sn by the internal oxide particle potentially contributed to the observed Sn enrichment. Such enrichment of a noble solute at metal/oxide interfaces due to the oxidation of the substrate and insolubility of the segregant in the oxide is well known—e.g., for Cu enrichment below the Fe-oxide scale during reheating [1,39,40]. For a mass-balance comparison, the number of segregated Sn atoms in the vertex regions, $I_{Sn}^{*}$, was determined for the selected six oxide particles with volumes larger than 100 nm^3^ via Equation (2):

(2) $$I_{Sn}^{*}=I_{Sn}-V_{Sn}\cdot C_{Sn}$$

where *I_Sn_* is the number of Sn ions within the Sn enrichment zone, *V_Sn_* the enrichment zone volume, and *C_Sn_* the nominal bulk concentration of Sn in atoms/nm^3^. In Figure 5e, the measured value of $I_{Sn}^{*}$ is compared to a theoretical number of enriched Sn ions, $I_{Sn}^{displ.}$, that would be expected when only the displacement of Sn by the growing oxide particle is considered, where $I_{Sn}^{displ.}$ is determined via the following equation:

(3) $$I_{Sn}^{displ.}=V_{Ox}\cdot C_{Sn}$$

where *V_Ox_* is the volume of the oxide particle. In this case, even with Sn assumed to be insoluble in the oxide and all Sn assumed to be transported to the vertex regions, $I_{Sn}^{displ.}$ can only account for 5–15% of $I_{Sn}^{*}$. Thus, displacement of Sn cannot explain the amount of Sn found in the vertex regions, suggesting that Sn enrichment occurs primarily as equilibrium segregation from the bulk to the interfaces of internal oxide particles. Consequently, the level of dissolved Sn in the matrix is decreased, limiting the Sn available for segregation to the metal/external oxide interface.

**Figure 6:**
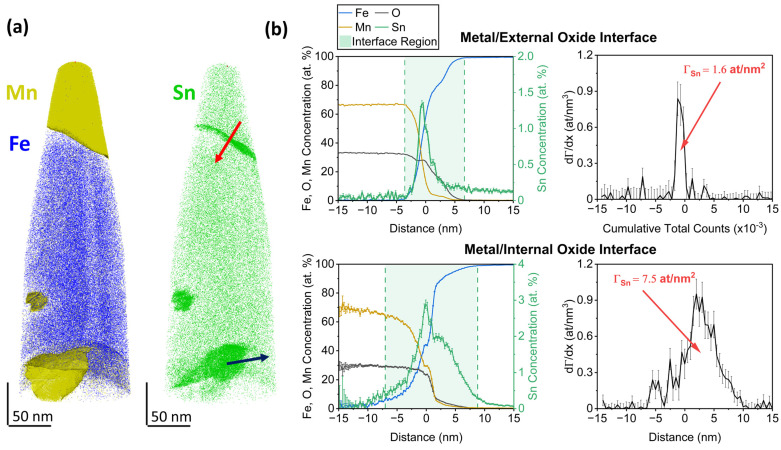
Quantification of Sn segregation to metal/oxide interfaces by APT. (**a**) Example of a reconstructed APT sample after annealing, Fe-10Mn-0.03Sn for 120 s at 825 °C. (**b**) Quantification of Sn segregation at the metal/oxide interfaces of an external (**top**) and internal (**bottom**) oxide along the red and blue arrows in (**a**), respectively. Elemental quantification is shown as concentration profile (**left**) and Sn interfacial excess Γ_Sn_ (**right**).

Figure 6 illustrates the quantification of interfacial Sn segregation from reconstructed APT data. The Mn, Fe, and Sn elemental maps (Figure 6a) qualitatively show Sn enrichment at the metal/oxide interfaces for the external and internal oxides, similar to the segregation observed at the external surface in Figure 5c. Concentration profiles normal to the interfaces were determined by proximity histograms based on the 50 at.% Fe iso-concentration surface. The inflection point of the Fe concentration profile as the principal matrix element was chosen for the origin [41,42,43]. The error bars represent the uncertainty in the measured concentration *s* based on counting statistics, where $s=\sqrt{\frac{X_{i}\left(1-X_{i}\right)}{n}}$, $X_{i}$ is the concentration of element *i,* and *n* is the total number of atoms in the analysis volume [44]. A strong peak in the Sn concentration profile (Figure 6b) can be observed in the interfacial regions, whose start and end (*x*_1_ and *x*_2_ integration limits in Equation (1), respectively) are marked by a change in the slope of the concentration profile of the principal elements [45,46]—i.e., Mn in the oxide and Fe in the metal substrate. Sn appears enriched over several nm, with maximum enrichment ratios of approximately 50 and 100 at the metal/oxide interfaces of the external and internal oxides, respectively. In this specific sample, both the maximum concentration and the interfacial excess are higher for the interface of the internal oxide than for the external oxide. Trends in the relative amounts of segregated Sn between the two metal/oxide interfaces as a function of annealing conditions could not be identified, as internal oxides were only captured in a subset of the APT specimens.

**Figure 7:**
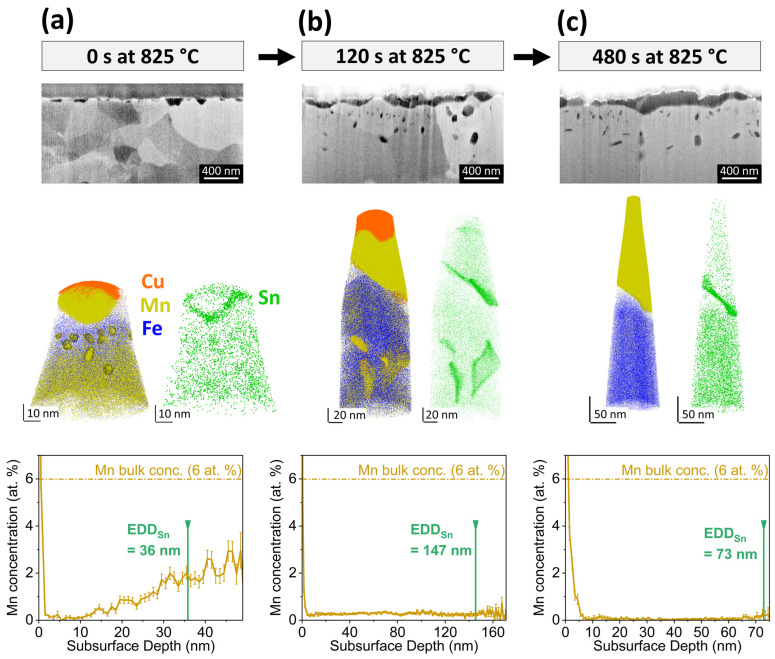
Evolution of metal/external oxide interface and Sn segregation layer during annealing. Cross-sectional SEM micrographs (**top**), APT elemental maps (**center**), and subsurface concentration profiles of solute Mn (**bottom**) for Fe-6Mn-0.03Sn isothermally annealed at 825 °C for (**a**) 0 s, (**b**) 120 s, and (**c**) 480 s. EDD_Sn_ indicates the equivalent depletion depth of Sn corresponding to the measured Sn interfacial excess in the individual sample.

The evolution of the oxide morphology and the Sn segregation layer during isothermal holding at 825 °C for Fe-6Mn-0.03Sn is shown in Figure 7. At the onset of isothermal holding, thin external oxides were present, some of which have grown into the subsurface (Figure 7a). No internal oxide particles were observed in the SEM micrographs. After 120 s, the external oxides have thickened and grown laterally, with partial coalescence into oxide patches (Figure 7b). Internal oxide particles can be observed on the subsurface of the sample. After 480 s at 825 °C, the thickness and coverage of the external oxides have further increased (Figure 7c). APT was used to closely track Sn segregation at the evolving metal/external oxide interface. In the elemental maps in Figure 7, the interface between Mn and Cu (the latter of which was used as a protective coating) is the original external surface of the oxide. For the isothermal holding times of 0 s and 120 s, the external oxides were thin enough to capture both the external surface and internal metal/oxide interface within one APT specimen. Sn segregation was observed at the metal/external oxide interface for all annealing conditions shown in Figure 7. However, no Sn enrichment at the external oxide surface or in the oxide itself was observed, which agrees with the AES results shown in Figure 3b. Thus, where the surface was covered with oxides, Sn that had segregated to the uncovered substrate surface during linear heating (Figure 3a) was now spread over the metal/external oxide interface.

Regarding internal oxidation, a cluster rich in Mn and O (represented by the 5 at.% Mn isosurfaces in Figure 7a) was concentrated within the first 30 nm of the subsurface at the onset of isothermal holding at 825 °C. After 120 s, Sn segregation to larger internal oxide particles could be observed. As particles coarsened over time, internal oxides were found less frequently in the immediate subsurface after annealing for 480 s.

As Mn oxides formed early during isothermal holding, the subsurface composition of the matrix was evaluated for Mn depletion. After internal oxide particles delineated by 10 at.% Mn iso-concentration surfaces were excluded, a concentration profile along the specimen longitudinal axis was measured to estimate the level of dissolved Mn in the matrix (bottom row of Figure 7). For all annealing conditions, Mn depletion was observed. After 0 s isothermal holding, no dissolved Mn was found in the first 10 nm of the subsurface. The Mn concentration increased with increasing distance from the interface from 0 to 2.6 at.% at the furthest point captured in this specimen. For increased holding times, the Mn depletion was more severe; the measured subsurface concentration of dissolved Mn did not exceed 0.5 at.%. For all holding times, the nominal Mn alloy concentration of 6 at.% was not reached within the distance from the interface captured by the APT specimens.

To estimate the origin of the Sn segregation, an equivalent depletion depth *EDD_Sn_* was determined for each of the specimens presented in Figure 7 via Equation (4):

(4) $$EDD_{Sn}=\frac{\Gamma_{Sn}}{C_{Sn}}$$

This metric represents a lower bound for the depth from which the segregated Sn originated, as ongoing Sn transport from deeper in the subsurface is not included. For all specimens shown in Figure 7, the *EDD_Sn_* was located within the Mn-depleted zone. Thus, the nominal bulk Mn composition of the system is not representative of the matrix chemistry in the region from which Sn segregated to the metal/external oxide interface. Instead, Sn segregated from a locale with a decreased Mn concentration.

**Figure 8:**
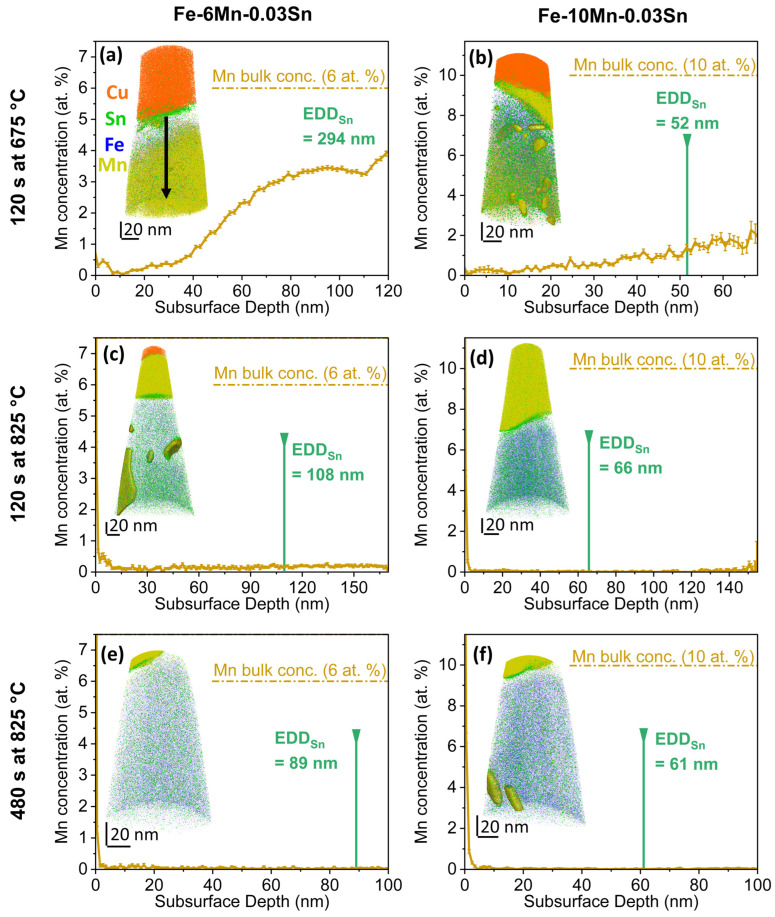
Near-surface Mn depletion during annealing. Elemental APT map and subsurface concentration profile of dissolved Mn in (**a**,**c**,**e**) Fe-6Mn-0.03Sn and (**b**,**d**,**f**) Fe-10Mn-0.03Sn after annealing for (**a**,**b**) 120 s at 675 °C, (**c**,**d**) 120 s at 825 °C, and (**e**,**f**) 480 s at 825 °C. EDD_Sn_ indicates the equivalent depletion depth of Sn corresponding to the measured Sn interfacial excess in the specific sample. Internal oxides are delineated by 10 at.% Mn isoconcentration surfaces. The concentration profiles were measured along the specimen longitudinal axis, as indicated by the arrow in (**a**).

The same trends in Mn depletion were confirmed for both the Fe-6Mn-0.03Sn and Fe-10Mn-0.03Sn alloys for all annealing conditions investigated by APT (Figure 8). The deviation of local subsurface chemistry from the nominal composition of the system, therefore, extends to lower temperatures within the CGL-compatible processing window. Comparing Figure 8a,b to Figure 8c,d and Figure 7a, Mn depletion was observed to increase with isothermal holding time and temperature, as would be expected [14].

**Figure 9:**
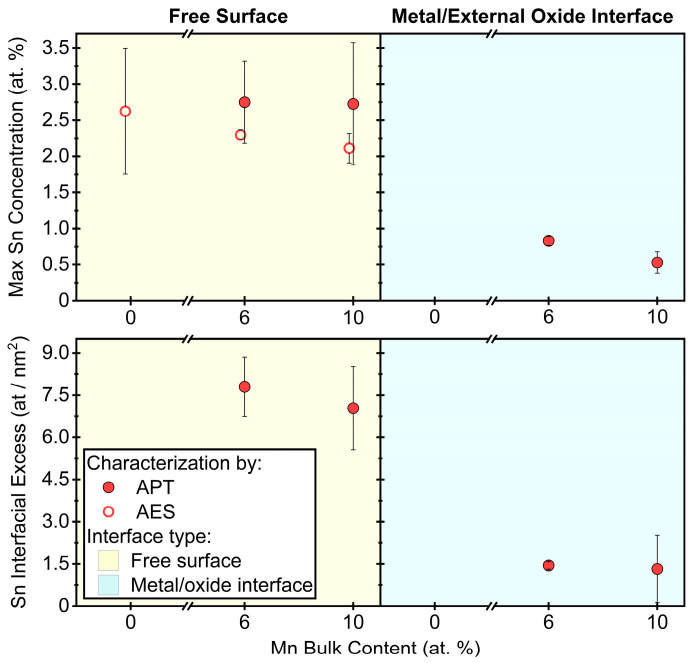
Interfacial Sn segregation after annealing for 120 s at 675 °C. Quantification of Sn segregation to the free surface between oxide nodules (**left**) and the metal/external oxide interfaces (**right**), as determined by APT and AES. Sn enrichment is expressed as maximum Sn concentration determined by a concentration profile across the interface (**top**) and as Sn interfacial excess (**bottom**).

**Figure 10:**
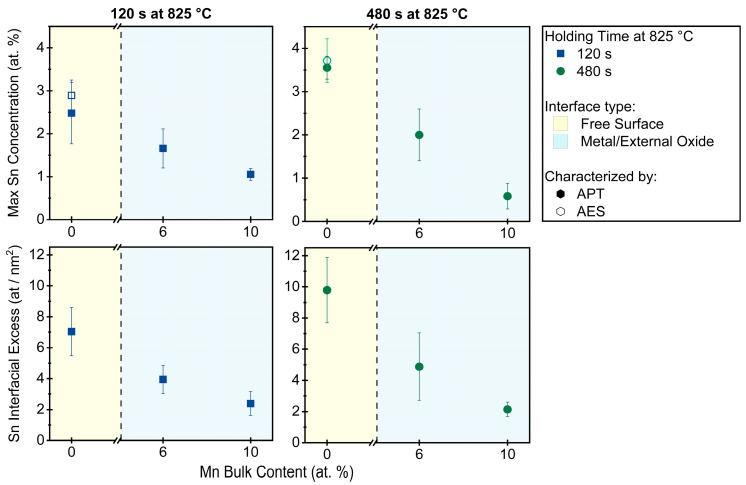
Interfacial Sn segregation at 825 °C. Quantification of Sn segregation to the free surface and the metal/external oxide interface after annealing at 825 °C for 120 s (**left**) and 480 s (**right**), as measured by APT and AES.

To compare the effect of the Mn concentration on the interfacial segregation of Sn between the free substrate surface and the metal/external oxide interface, Sn enrichment was quantified by APT for Fe-0.03Sn, Fe-6Mn-0.03Sn, and Fe-10Mn-0.03Sn annealed for 120 s at 675 °C, 120 s at 825 °C, and 480 s at 825 °C. These data are summarized in Figure 9 and Figure 10, where the values are based on 32 data sets acquired by APT. For all samples, significant Sn segregation was observed. Trends of Sn segregation as a function of Mn level are reflected in both metrics for interfacial Sn (maximum Sn concentration and interfacial excess) and agree with the AES data reported in Figure 3a.

Figure 9 shows the maximum Sn concentration and the interfacial excess for samples annealed for 120 s at 675 °C. For this condition, the oxide coverage was sufficiently low to capture both the external oxides and internodular space within one FIB lift out. For each interface type, the amount of segregated Sn did not vary significantly with substrate Mn content. However, the Sn segregation to the metal/external oxide interface was significantly lower than that to the free substrate surface for all Mn levels for this annealing condition (Figure 9).

Sn segregation after annealing at 825 °C is shown in Figure 10. For the Mn-containing grades, all samples exhibited significant external oxidation with high area fractions covered by oxides. For both isothermal holding times of 120 s and 480 s, less Sn was found at the metal/external oxide interface than at the free surface, similar to what was observed at 675 °C. In addition, decreased Sn segregation to the metal/external oxide interfaces with increasing alloy Mn was observed (Figure 10). More Sn was found at the metal/external oxide interfaces for the 825 °C (Figure 10) than for the 675 °C (Figure 9) annealing conditions.

### 3.3. Thermodynamic and Oxide Morphology Effects on Sn Segregation

**Figure 11:**
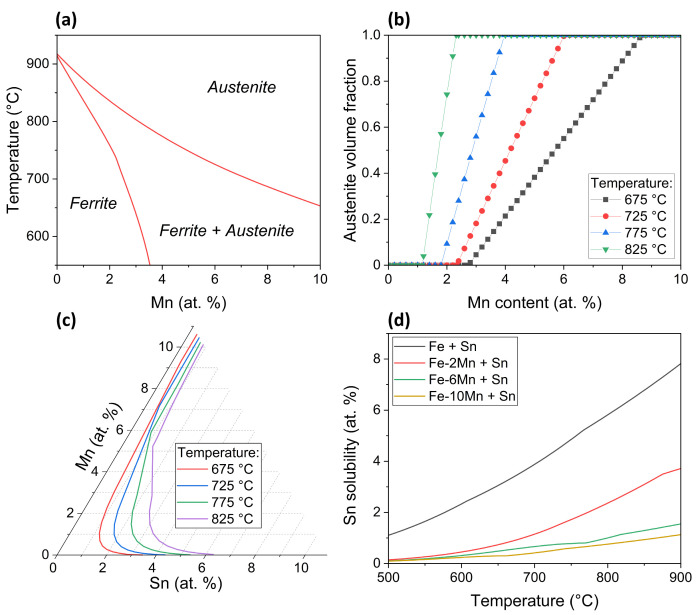
Chemistry-related effects of Mn addition. (**a**) Section of the Fe-Mn-0.03Sn phase diagram for the investigated isothermal holding temperatures and (**b**) derived volume fraction of austenite as a function of temperature and Mn alloy content. (**c**) Sn solvus line in the Fe-rich corner of the Fe-Mn-Sn ternary phase diagram and (**d**) Sn solubility in Fe-(0–10 at.%)Mn as a function of temperature.

Important process variables governing interfacial segregation are the solubility of the segregant and its diffusivity, which are strong functions of the phases present in the matrix. Thermo-Calc Software 2022a with the TCFE12 database [47] was used to calculate the phase diagram shown in Figure 11. Figure 11a shows an isopleth of the Fe-Mn-0.03Sn phase diagram corresponding to the investigated thermal treatment window. Depending on the annealing temperature and the Mn concentration, the matrix is ferrite, austenite, or a mixture of both. The austenite volume fraction as a function of alloy Mn content is shown in Figure 11b, which (as expected) shows that with increasing temperature, the austenite volume fraction increases rapidly with increasing alloy Mn content. For all temperatures investigated (Table 2), the matrix was ferritic for the 0 at.% Mn addition and fully austenitic for the 10 at.% Mn alloy. The solvus line of Sn in the Fe-rich corner of the Fe-Mn-Sn ternary phase diagram and the Sn solubility as a function of temperature and Mn content are shown in Figure 11c and Figure 11d, respectively. At all temperatures, it can be seen that the Sn solubility decreases with increasing alloy Mn concentration.

**Figure 12:**
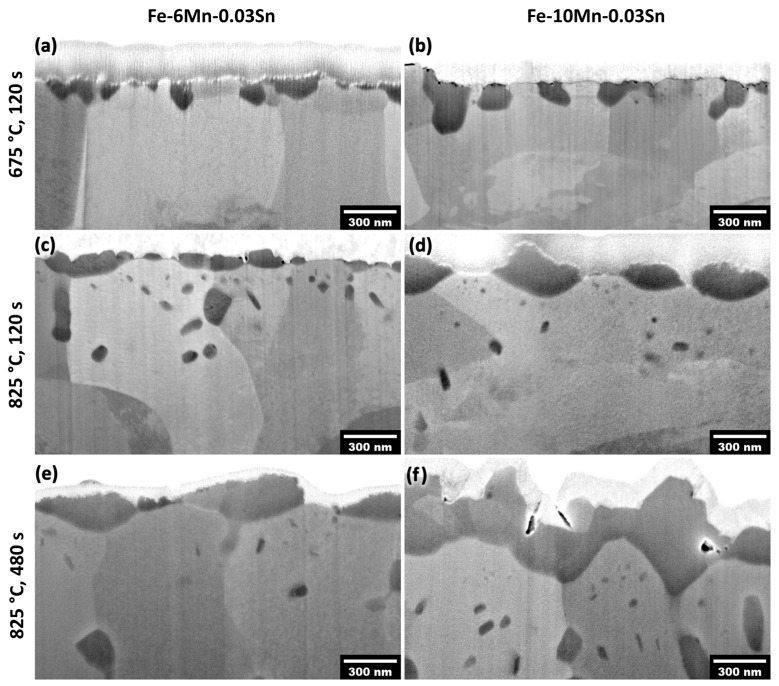
Morphology of internal and external oxides. Cross-sectional SEM micrographs of (**a**,**c**,**d**) Fe-6Mn-0.03Sn and (**b**,**d**,**f**) Fe-10Mn-0.03Sn after annealing for (**a**,**b**) 120 at 675 °C, (**c**,**d**) 120 s at 825 °C, and (**e**,**f**) 480 s at 825 °C. The light gray metal layer on top of the darker oxides is part of the protective W deposit.

**Figure 13:**
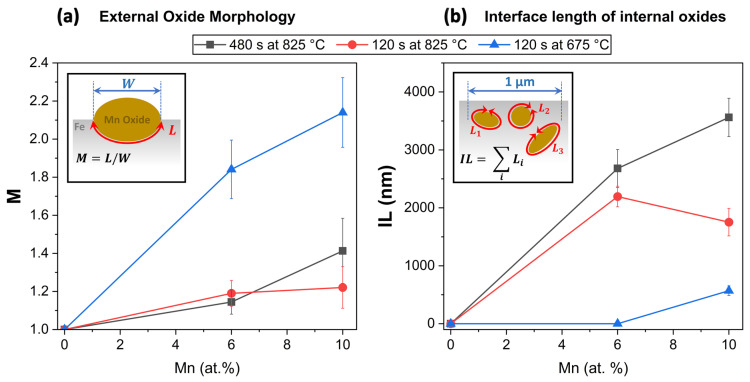
(**a**) Characterization of external oxide nodules subsurface morphology and (**b**) total interface length of internal oxide particles.

Furthermore, the alloy Mn concentration affected the extent of selective oxidation and the oxide morphology (see Figure 12). This, in turn, affects the relative interfacial areas at the free substrate surface, metal/external oxide interfaces, and metal/internal oxide interfaces, which are documented in Figure 13. For example, after annealing for 120 s at 675 °C, individual oxide nodules were present on the surface (Figure 12a,b). In this case, oxide growth occurred primarily from the original surface into the subsurface region along grain boundaries. The oxide size and penetration depth were higher for the Fe-10Mn-0.03Sn alloy (Figure 12b) compared to the Fe-6Mn-0.03Sn alloy (Figure 12a) for the same annealing condition, while only a few internal oxide particles were observed. When annealed at 825 °C, the external oxide nodules grew both inwards and thickened with a larger number of internal oxides being observed (Figure 12d–f). The external oxides were larger with increased Mn level after holding for 120 s and 480 s at 825 °C (Figure 12c,e vs. Figure 12d,f).

The subsurface morphologies of the external oxide nodules were described by the parameter M=WL, where *W* is the measured length of the metal/external oxide interface and *L* is the width of the oxide (see Figure 13). *M* expresses the factor by which the length of the interface is increased due to the presence of the inward-growing oxide. Considering the Sn segregation to the free substrate surface before oxide formation, it also represents the factor by which the concentration of the pre-segregated Sn is decreased due to the spread over a larger interfacial area. *M* = 1 indicates an uncovered surface or the interface of an oxide nodule without inward growth, while an oxide with a hemispherical subsurface morphology is described by *M* = 2. Where oxide particles coalesced into patches, an effective value for each 1 µm wide segment of the cross section was determined. The parameter *M* and the total interface length of internal oxides, *IL*, as a function of the annealing condition and Mn alloy concentration are shown in Figure 13.

After annealing for 120 s at 675 °C, the external oxides showed an *M* value of approximately 2 (Figure 13a) for both the 6Mn and 10Mn alloys. Due to the small number of internal oxides (Figure 12a,b), the total interface length of the internal oxides was low compared to the other annealing conditions (Figure 13b). The difference between Sn at the free surface and Sn at the metal/external oxide interface reported in Figure 9 can, therefore, be attributed primarily to the external oxide morphology and the associated spread of the pre-segregated Sn over an increased interfacial area.

At 825 °C, the stronger lateral growth of the oxide particles (Figure 12c–f) is reflected in lower *M* values compared to the 675 °C annealing condition (Figure 13a). Thus, spreading of the pre-segregated Sn alone cannot account for the decreased Sn levels of the different interface types seen in Figure 10. However, the high density of internal oxides for the 825 °C annealing condition (Figure 12c–f) resulted in a significant increase in additional internal interfacial area, with *IL* values of 1.5–3.7 µm/µm width of the cross section (Figure 13b). The overall interfacial length was, therefore, more than doubled by the presence of internal oxides. As discussed in Section 3.2, the metal/internal oxide interfaces are also sites of Sn segregation (Figure 5 and Figure 6). Acting as Sn sinks, they decrease the amount of dissolved Sn available for ongoing segregation of Sn to the metal/external oxide interface. Overall, the difference in Sn enrichment at the free substrate surface vs. metal/external oxide interface observed after annealing for 120 and 480 s at 825 °C (Figure 10) can be explained by the combined effect of the external oxide morphology and the presence of internal oxide particles.

## 4. Discussion

During selective oxidation of Fe-(0–10)Mn-0.03Sn (at.%) alloys, Sn was observed to segregate to external and internal interfaces. While the Sn enrichment at the external surface was unaffected by the Mn concentration of the alloy (Figure 3a and Figure 9), the Sn concentration at the metal/external oxide interface decreased with increasing Mn alloy content (Figure 9 and Figure 10).

**Figure 14:**
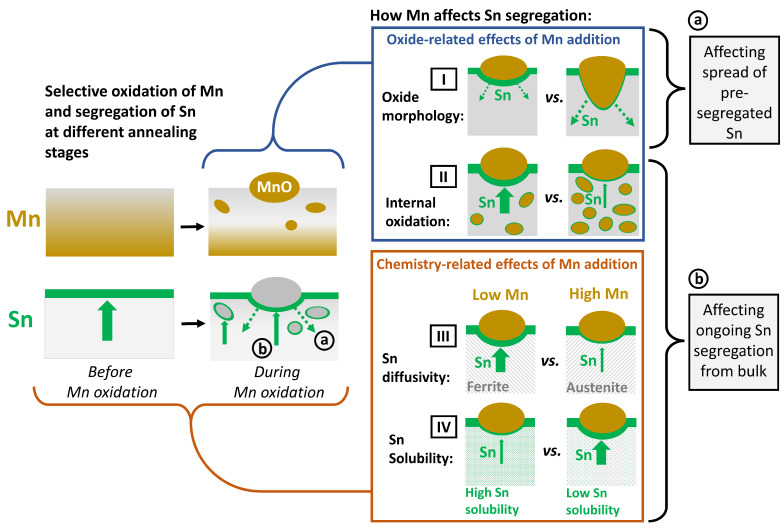
Schematic of mechanism for the effects of Mn addition on interfacial segregation of Sn.

Figure 14 illustrates the selective oxidation and Sn segregation processes at the different stages of the annealing cycle and how they are affected by the Mn concentration of the substrate. At the early stages of linear heating (see schematic in Figure 1), Sn segregates strongly to the surface before significant coverage by external oxides is established (Figure 2a–c and Figure 3a). As selective oxidation progresses, internal oxides form and external oxide nodules grow—i.e., thickening, growing laterally, and growing into the substrate along GBs (Figure 2 and Figure 12)—such that an increasing fraction of the free surface is converted to a metal/oxide interface. Mn is bound in Mn oxides, and the immediate subsurface is depleted of solute Mn (Figure 7 and Figure 8). The pre-segregated Sn, established during linear heating to the peak annealing temperature, is now distributed over the increased interfacial area between the matrix and oxide (Figure 13a and (a) in Figure 14). Sn segregation from the bulk to the metal/oxide interfaces of both external (Figure 9 and Figure 10) and internal oxides (Figure 4) continues (marked (b) in Figure 14).

Interfacial segregation of Sn in Fe-based substrates is commonly modeled by an equilibrium segregation mechanism [36,48,49], the main driving force for which is the reduction in interfacial energy and the release of elastic strain energy due to the size misfit of the solute Sn with respect to the matrix atoms. As this driving force is larger for the free external surface than for internal interfaces [50,51], the rate of segregation to metal/oxide interfaces is expected to be lower than for the uncovered surface at initial stages of oxidation. Overall, the total amount of Sn enriched at the metal/external oxide interface is the combined result of the two processes illustrated in Figure 14a,b.

Factors that increase the tendency to segregate are also known to decrease the solubility limit of the segregant in the matrix [6,52,53,54]. Such an inverse relationship between the enrichment level and solubility is reflected in the truncated BET isotherm for interfacial segregation in dilute binary alloys [55,56]:

(5) $$\frac{\theta_{\infty }}{\theta_{\infty }^{0}-\theta_{\infty }}=\frac{X}{X^{*}}\exp\left(-\frac{\Delta G}{RT}\right)$$

where $\theta_{\infty }$ is the equilibrium surface coverage and $\theta_{\infty }^{0}$ the saturation coverage of the segregant on the substrate. *X* denotes the bulk atomic fraction, X^*^ the solid solubility, and Δ*G* the free energy of segregation of the segregant in the substrate.

This concept has successfully been expanded to multi-component systems: within the framework of the Guttmann theory for interactive segregation, the increased segregation of P by the addition of Ni, Mn, and Cr was explained by their effect of lowering P solubility [57]. More recently, Mn was found to enhance Sb segregation by reducing its solubility in Fe [45]. For the present case, Mn decreases the solubility of Sn in Fe (Figure 11c,d); thus, enhanced surface segregation is predicted based on the BET isotherm. However, the enrichment of Sn at the free surface between oxide nodules is insensitive to the Mn level of the substrate (Figure 3 and Figure 9), which can be rationalized considering the effect of Mn on the diffusivity of Sn in Fe. The kinetics of surface segregation can be described by [48]:

(6) θ(t)=θ∞1−expDtα2d2erfcDtα2d212

where $\theta \left(t\right)$ is the transient surface concentration, *D* is the bulk diffusivity of the segregant in the matrix, *d* is the thickness of the enrichment layer, and α is the steady-state enrichment ratio $\theta_{\infty }/X$. A first-order approximation of Equation (6)

(7) θt=2XDtπd212

illustrates that the surface concentration scales parabolically with *D* at the early stages of segregation.

For the annealing conditions investigated, Mn increases the volume fraction of austenite in the matrix (Figure 11a,b). As is the case with other substitutional solutes in Fe, such as Co, Mn, Ni, and Cu [58], the diffusion of Sn is significantly slower in austenite (γ-Fe) than in ferrite (α-Fe). The ratio of the Sn diffusivities can be estimated as $\frac{D_{Sn}^{\gamma -Fe}}{D_{Sn}^{\alpha -Fe}}\approx 0.007$ at the allotropic temperature of Fe [59,60]. Thus, the thermodynamic effect of Mn additions (increased $\theta_{\infty }$ due to reduced Sn solubility, marked [IV] in Figure 14) is to some degree compensated by the kinetic effect of Mn on the substrate phases (reduced *D* due to increased volume fraction of austenite, marked [III]).

During annealing, the oxidation reaction between Mn and O leads to the formation of Mn oxides, which precipitate once the oxide solubility limit in Fe is exceeded, where oxide precipitation leads to Mn and O depletion in the vicinity of the oxide particles (Figure 7 and Figure 8). Based on the models for interactive equilibrium segregation discussed above (e.g., the Guttmann model), Sn segregation is expected to be affected by the elements dissolved in the Fe matrix [57]. Thus, an additional factor contributing to the observed insensitivity of Sn surface segregation to the substrate Mn level is the subsurface depletion of Mn. As selective oxidation progresses, the subsurface regions in which Sn segregation occurs become chemically similar, irrespective of the initial Mn concentration. The effect of the different Mn contents of the Fe-xMn-0.03Sn alloys is, therefore, less than that predicted from the nominal bulk composition of the substrate according to Equation (5). Similarly, the effect of the O concentration at the metal/oxide interface on Sn segregation via direct chemical interaction is not expected to be the decisive factor in determining Sn enrichment at metal/oxide interfaces compared to the free surface, as O is bound in the oxide.

The chemical effects of Mn additions discussed so far cannot explain the decreased Sn segregation with increased Mn levels observed at the metal/external oxide interfaces. In this case, a portion of the Sn at the metal/external oxide interface originates from the Sn segregated to the surface before it was covered by oxides (Figure 14a). As shown schematically in Figure 14 (marked [I]) and calculated in Figure 13, the oxide morphology determines the interfacial area over which this pre-segregated Sn is spread. The morphology of an external oxide nodule can be described by the parameter *M* (Figure 13a), which is a proxy for the ratio between the area of the metal/external oxide interface and the base area of the oxide particle (i.e., the area of the original free surface that was converted to a metal/oxide interface). During the inward growth of a high-*M* oxide, the pre-segregated Sn is now distributed over a larger interfacial area, resulting in decreased Sn enrichment compared to a shallower, low-*M* particle (Figure 12 and Figure 13a).

Ongoing Sn segregation to metal/oxide interfaces was observed after significant oxide coverage had been established (Figure 5 and Figure 6). The Sn enrichment around internal oxide particles was shown to originate from the bulk, as discussed in Section 3.2 (Figure 5). Consequently, the amount of dissolved Sn in the subsurface region is more strongly reduced by the larger total interfacial area of the internal oxides, as was observed with increasing isothermal holding time and temperature (Figure 12b). This results in a reduction of the effective concentration of Sn available for segregation to the metal/external oxide interface. Equations (5) and (7) show how the interfacial Sn concentration scales approximately linearly with the Sn bulk concentration both at equilibrium and at the early stages of segregation. Thus, the ongoing segregation from the bulk to the metal/external oxide interface is decreased (marked [II] in Figure 14) with the decrease in the effective Sn bulk concentration. Overall, it was demonstrated that an evaluation of the effect of Mn on Sn segregation cannot be based on chemical effects alone; the characteristics of the oxides that form by the selective oxidation of Mn and their associated metal/oxide interfaces must also be considered. The implications of the Mn-Sn interaction for the suitability of Sn microalloying for decreasing selective oxidation kinetics and enhancing reactive wetting of Fe-Mn-Sn alloys will be subjects of subsequent publications.

## 5. Conclusions

The effects of the Mn concentration on the interfacial segregation of Sn during CGL-compatible annealing were investigated for a series of Fe-(0–10)Mn-0.03Sn (at.%) model alloys. The following conclusions can be drawn:

1. Significant Sn enrichment at the external substrate surface and at internal metal/oxide interfaces was observed for isothermal holding at 675–825 °C for 0–480 s and for Mn alloy contents of 0–10 at.%.
2. Sn segregation to the free surface between oxide nodules was not significantly affected by the Mn concentration of the substrate. Equilibrium segregation models predicted an ambivalent chemistry-related effect of Mn concentration on Sn segregation, contributing to the insensitivity of Sn segregation to the Mn content of the alloy. In the present case, Mn reduces the Sn solubility in Fe and, therefore, increases the driving force for Sn segregation. However, Mn also increases the volume fraction of austenite, thereby reducing the effective Sn diffusivity and decreasing the segregation kinetics.
3. Selective oxidation led to a depletion of solute Mn near the metal/external oxide interface, making the subsurface chemically similar between the different alloys. The global composition of the substrate is, therefore, not an adequate proxy to describe the local chemistry of the region in which Sn segregation occurs.
4. Sn enrichment at the metal/external oxide interface was lower than at the free surface and decreased with increasing alloy Mn concentration. This is attributed to differences in the subsurface morphologies of external oxides and the interfaces of internal oxide particles acting as Sn sinks.
5. The observed Sn segregation is promising for a potential industrial application of Sn microalloying to improve galvanized coatings in a wide range of compositions and processing conditions typical of CGL-compatible AHSSs. Suitability for individual steel grades cannot be evaluated based on global composition alone but must consider all factors determining oxide characteristics, such as the annealing atmosphere and temperature, which will be investigated in detail in future work.

## Figures and Tables

**Table 1:** Compositions of Fe-Mn-Sn model alloys.

Alloy System	Mn Concentration (at.%)	Sn Concentration (at.%)
Fe-0.03Sn	0.01	0.034
Fe-2Mn-0.03Sn	1.97	0.035
Fe-6Mn-0.03Sn	6.05	0.034
Fe-10Mn-0.03Sn	9.85	0.034

**Table 2:** Parameters of experimental annealing heat treatments and experimental matrix for quantification of Sn segregation by Auger Electron Spectroscopy (AES) and Atom Probe Tomography (APT).

Peak Annealing Temperature—PAT (°C)	Oxygen Partial Pressure—pO_2_ (atm)	Isothermal Holding Times—t (s)	
		Surface Segregation Quantified by AES	Interfacial Segregation Quantified by APT
675	9.2 × 10^−25^	0, 60, 120, 240, 480	120
725	2.2 × 10^−23^	0, 60, 120, 240, 480	
775	3.7 × 10^−22^	0, 60, 120, 240, 480	
825	5.0 × 10^−21^	0, 60, 120, 240, 480	0, 120, 480

## Data Availability

The raw data supporting the conclusions of this article will be made available by the authors on request.
